# COVID-19-Associated Fungal Infections: An Urgent Need for Alternative Therapeutic Approach?

**DOI:** 10.3389/fmicb.2022.919501

**Published:** 2022-06-09

**Authors:** Marianna Domán, Krisztián Bányai

**Affiliations:** ^1^Veterinary Medical Research Institute, Budapest, Hungary; ^2^Department of Pharmacology and Toxicology, University of Veterinary Medicine, Budapest, Hungary

**Keywords:** fungal co-infections, *Candida auris*, COVID-19-associated aspergillosis, antimicrobial peptide, COVID-19-associated mucormycosis

## Abstract

Secondary fungal infections may complicate the clinical course of patients affected by viral respiratory diseases, especially those admitted to intensive care unit. Hospitalized COVID-19 patients are at increased risk of fungal co-infections exacerbating the prognosis of disease due to misdiagnosis that often result in treatment failure and high mortality rate. COVID-19-associated fungal infections caused by predominantly *Aspergillus* and *Candida* species, and fungi of the order *Mucorales* have been reported from several countries to become significant challenge for healthcare system. Early diagnosis and adequate antifungal therapy is essential to improve clinical outcomes, however, drug resistance shows a rising trend highlighting the need for alternative therapeutic agents. The purpose of this review is to summarize the current knowledge on COVID-19-associated mycoses, treatment strategies and the most recent advancements in antifungal drug development focusing on peptides with antifungal activity.

## Introduction

Fungal diseases remain a significant medical issue considering as a worldwide threat to human health affecting close to one billion individuals. There are several reasons for ongoing increase of invasive fungal infections, including the use of immunosuppressive therapies in context of cancer treatment or transplantation, the increased use of modern medical devices, such as catheters and implants, and the use of broad-spectrum antibiotics. The coronavirus disease 2019 (COVID-19) pandemic further worsened the current situation since this viral respiratory disease predispose the patients to secondary life-threatening fungal infections in the intensive care units (ICUs) making adequate diagnosis more difficult. The overlapping respiratory manifestations complicate the treatment of COVID-19. Prolonged hospitalization period and potentially required mechanical ventilation along with lymphopenia, leukopenia, and systemic hyperinflammatory reaction facilitates the growth of fungi in COVID-19 patients (Arastehfar et al., 2020a; Amin et al., 2021; Baddley et al., 2021; Gregoire et al., 2021). In addition, World Health Organization (WHO) COVID-19 treatment guideline recommend empirically prescribed broad-spectrum antibiotics to treat possible bacterial co-infections, but only in severe COVID-19 patients (World Health Organization, 2021). The main causative organisms responsible for the majority of serious fungal diseases are *Candida, Aspergillus, Mucorales*, and *Cryptococcus*. Despite of fungal healthcare-associated invasive infections have unacceptably high mortality rate, the number of deaths is most likely an underestimation due to poor epidemiological data and misdiagnosis (Bongomin et al., 2017). The high clinical mortality and economic burden posed by invasive fungal infections has resulted in the widespread use of antifungal agents. Antifungal treatments and/or prophylaxis are essential to reduce comorbidities and mortalities caused by fungal infections. However, due to selective drug pressure, the efficacy of the limited systemic antifungal drugs has been changed yielding species with less predictable antifungal susceptibility (Friedman and Schwartz, 2019). Microbial resistance is common in certain fungal species and involves both intrinsic resistance (strains are inherently less susceptible to a given antifungal agent), and secondary resistance (acquired resistance in an otherwise susceptible strain following drug exposure). The most notable species emerging worldwide and regarded as major concern for public health are triazole-resistant *Aspergillus fumigatus* (Verweij et al., 2016; Romero et al., 2019; Yang et al., 2021), *C. tropicalis, C. parapsilosis* (Pfaller et al., 2019), multidrug-resistant (MDR) *Candida auris* (Chowdhary et al., 2018; Chaabane et al., 2019; Chow et al., 2020) and MDR *C. glabrata* showing increasing prevalence globally (Haeley and Perlin, 2018). The narrow spectrum activity and cross-resistance due to similar mechanisms of action across drugs has triggered the search for safer alternatives with reduced toxicity, improved pharmacodynamics and pharmacokinetics, and increased specificity (Parente-Rocha et al., 2017; Gintjee et al., 2020; Duncan et al., 2021). In this review we provide an overview of emerging fungal diseases in COVID-19 patients highlighting the current antifungal treatments. We also highlight some aspects of new antifungal drug development with a great promise to overcome resistance issues. In particular, we focus on novel antifungal peptides as other emerging alternatives, such as agents with new structure for a known target or entirely novel targets, combination therapy of antifungals with non-antifungal drugs and quorum-sensing molecules have been recently reviewed by other authors (Kovács and Majoros, 2020; Bouz and Doležal, 2021; Rossato et al., 2021).

### Candida auris

In the last decade, a multidrug-resistant nosocomial pathogen, *C. auris*, has emerged and spread worldwide. Since its discovery in 2009 from the ear discharge of a Japanese patient (Satoh et al., 2009), *C. auris* has been reported from 46 countries causing outbreaks in healthcare institutions (Allaw et al., 2021; Chakrabarti and Sood, 2021; de Almeida et al., 2021; Kurt et al., 2021; Pandya et al., 2021; Shaukat et al., 2021). Phylogenetic analysis using whole genome sequencing has revealed deep divergence within the *C. auris* species. High inter-clade genetic diversity has been found suggesting that distinct clades emerged independently at different geographic regions (Lockhart et al., 2017). Four major clades have been described so far: South Asian (clade I), South African (clade III), South American (clade IV) and East Asian (clade II). A potential fifth clade has also been identified in Iran (Chow et al., 2019; Rhodes and Fisher, 2019). The majority of strains in the East Asian clade was isolated from ear infection only, whereas other clades are known to cause nosocomial invasive infections and outbreaks in healthcare settings (Welsh et al., 2019).

Invasive microbial co-infections during hospitalization may lead to more severe outcomes. The crude in-hospital mortality rate of invasive infections caused by *C. auris* is larger than that of caused by other *Candida* species, ranging from 30 to 72% (Taori et al., 2019; Arensman et al., 2020; Chakrabarti and Singh, 2020; de Almeida et al., 2021; Garcia-Bustos et al., 2021). *C. auris* superinfections in critically ill COVID-19 patients have been associated with 30-day mortality rates usually above 50%, although the high case-fatality rate is multifactorial and not exclusively attributed to *C. auris* infection (Table 1; Chowdhary et al., 2020; Magnasco et al., 2021; Villanueva-Lozano et al., 2021).

**TABLE 1:** Comparison of the characteristics of COVID-19-associated fungal infections.

Fungal infection	Cohort size	Identification/diagnosis	Risk factors and comorbidities	Co-infections	Antifungal treatment	Outcome	References
*Candida auris*	10	Culture	Hypertension, diabetes mellitus, chronic kidney and liver disease	Bacteremia in 4 patients: *Enterobacter cloacae, Staphylococcus haemolyticus*	NA	6 died	Chowdhary et al., 2020
	2	Culture positivity by Vitek 2	Mechanical ventilation, hemodialysis, CVC; chronic renal insufficiency, diabetes mellitus, hypertension	CVC culture: MDR *Enterococcus faecalis*; catheter-related BSI: carbapenem-resistant *Acinetobacter baumannii*; and carbapenem-resistant *Morganella morganii, Klebsiella pneumoniae*	Empirical ANI or ANI	1 died	de Almeida et al., 2021
	12	Blood and urine culture	Mechanical ventilation, high blood pressure, diabetes mellitus type 2, obesity, coronary artery disease, acute kidney injury, hypothyroidism, valvular heart disease, asthma	In 10 patients: *Pseudomonas aeruginosa, K. pneumoniae, Candida glabrata, E. faecalis, Stenotrophomonas maltofilia*, Cytomegalovirus	CAS, ANI, ISZ, VOR, AMB	8 died	Villanueva-Lozano et al., 2021
	4	Cultured from blood and identified by MALDI-TOF MS	Coronary artery disease, hypertension, asthma	Carbapenem-resistant *P. aeruginosa*	CAS, AMB	2 died	Magnasco et al., 2021
	4	Cultured from blood, urine, deep tracheal aspirate and identified by MALDI-TOF MS	Metastatic prostate cancer, cutaneous T cell lymphoma in remission, chronic lymphocytic leukemia	NA	CAS therapy of 3 patients	No patients died at the time of publication	Allaw et al., 2021
Aspergillosis	9	EORTC-MSG criteria and GM in BAL, serum; 8 case putative CAPA, 1 case probable CAPA	Myeloma, steroids	NA	VOR, CAS therapy of 2 patients	4 died	Alanio et al., 2020
	5	*AspICU* algorithm and GM in BAL or serum; putative CAPA	Arterial hypertension, diabetes mellitus, chronic obstructive pulmonary disease, obesity, hypercholesterolemia, steroids	Human metapneumovirus	VOR, ISZ, CAS	3 died	Koehler et al., 2020
	5	Clinical signs and symptoms, an abnormal lung imaging, respiratory specimen culture positive for *Aspergillus* spp., GM in BAL or serum; putative CAPA	Diabetes mellitus, steroid and tocilizumab therapy	*K. pneumoniae, P. aeruginosa, MRSA, S. maltophilia*, MDR *Acinetobacter* spp.	AMB, VOR	3 died (the cause of death was ARDS)	Nasir et al., 2020
	6	Culture and GM in BAL; 3 possible and 3 probable CAPA	Cardiomyopathy, chronic obstructive pulmonary disease, corticosteroid therapy, asthma	NA	VOR + ANI combination, AMB	4 died	van Arkel et al., 2020
	3	Culture, serum GM, serum BDG; 2 putative and 1 probable CAPA	Hypertension, diabetes mellitus type 2, pulmonary fibrosis, obesity, asthma, antibacterial therapy, tocilizumab therapy	*Haemophilus influenzae*	VOR	1 died	Lamoth et al., 2020
	7	*AspICU* algorithm, 4 proven CAPA cases	Obesity, hypercholesterolemia, arterial hypertension, diabetes mellitus, chronic kidney disease, acute myeloid leukemia, mechanical ventilation	NA	VOR, ISZ therapy in 6 patients	4 died	Rutsaert et al., 2020
	6	Clinical, radiological data, GM (from serum and sputum), tracheal or bronchial culture, GM test from BAL; probable CAPA; post-mortem histopathology-no fungi were observed	Chronic kidney injury, diabetes mellitus; hypertension, corticosteroid therapy	NA	VOR + ANI combination	6 died	Flikweert et al., 2020
Mucormycosis	11 (3 mucormycosis co-infecton + 8 post-COVID-19 mucormycosis)	Culture and histopathological examination	Diabetes mellitus, hypertension, hypothyroidism, renal transplant, chronic kidney disease	*K. pneumoniae, P. aeruginosa, Serratia marcescens*	AMB; sinus surgery with debridement in 7 patients	6 died (2 died despite surgery)	Kamath et al., 2022
	4	Culture	Diabetes mellitus, obesity, chronic lymphocytic leukemia, steroid therapy, mechanical ventilation	*Aspergillus fumigatus*	AMB, POS, CAS, ISZ (2 patients received no antifungals), surgical debriment	3 died (1 no information)	Buil et al., 2021
	8 (1 case + literature review)	Culture and biopsy	Diabetes mellitus, hypertension, chronic kidney disease, hypothyroidism, asthma, obesity, corticosteroid use	*M. morganni, E. cloacae*, vancomycin-resistant *Enterococcus faecium, Klebsiella variicola*, CNS	AMB, POS, CAS, ISZ (2 patients received no antifungals)	8 died	Khatri et al., 2021
	17	Culture, biopsy, histology; 5 proven, 12 probable CAM	Diabetes mellitus, hematological malignancy, chronic obstructive pulmonary disease, corticosteroid use	*Aspergillus* spp.	AMB, ISZ (five patients received no antifungals)	15 died	Danion et al., 2022

*AMB, amphotericin B; ANI, anidulafungin; ARDS, acute respiratory distress syndrome; BAL, bronchoalveolar lavage; BDG, 1,3-β-D-glucan; CAM, COVID-19-associated mucormycosis; CAPA, COVID-19-associated aspergillosis; CAS, caspofungin; CNS, coagulase-negative Staphylococcus; CVC, central venous catheters; EORTC-MSG, European Organization for research and treatment of cancer mycoses study group; GM, galactomannan; ISZ, isavuconazole; MDR, multidrug resistant; MICA, micafungin; MRSA, methicillin-resistant Staphylococcus aureus; NA, not available; POS, posaconazole; VOR, voriconazole.*

The pandemics of COVID-19 has overwhelmed healthcare facilities promoting the nosocomial transmission of *Candida* species especially in ICUs. Both prospective and retrospective data of COVID-19 patients admitted to ICU suggested that risk factors driving the high incidence of candidaemia include prolonged hospital stay, mechanical ventilation, central venous catheters, surgical procedure, and use of broad-spectrum antibiotics, steroids and immunosuppressant drugs (Ezeokoli et al., 2021; Nucci et al., 2021). The hospital environment also plays a crucial role in the local spread of *C. auris* as it is able to efficiently survive for 7 days on steel and porous surfaces and for 14 days on plastics. *C. auris* survives desiccation and resists quaternary ammonium compound disinfectants, peracetic acid, standard ultraviolet-C cycle times and standard concentration of sodium hypochlorite as well (Welsh et al., 2017; Cadnum et al., 2018; Rutala et al., 2019; Chakrabarti and Sood, 2021). Healthcare workers and physicians transiently colonized on their hands, nares and groin, and contaminated medical devices may facilitate the dissemination of *C. auris*. Nobrega de Almeida et al. (2021) investigated *C. auris* colonization of patients, healthcare workers, and inanimate sites of a Brazilian hospital. Among the samples collected from inanimate surfaces, the digital thermometers had the highest rate of positive cultures (17%), followed by bed rails (14.9%), vital signs monitors/intravenous infusion pumps (10.6%), and tray tables (10.6%). In addition, the COVID-19 pandemic may provide ideal conditions for prolonged outbreaks of *C. auris* in hospital ICUs due to over-occupancy and limited resources for infection control practices (e.g., prolonged usage of personnel protective equipment through shortages) (Chowdhary et al., 2020; Allaw et al., 2021; Prestel et al., 2021; Villanueva-Lozano et al., 2021). However, observational studies showed that *C. albicans* and *C. glabrata* and not *C. auris* were the causative agent in the majority of COVID-19-associated candidaemia significantly increasing the mortality rate of patients as well (Casalini et al., 2021). Machado et al. reported that the incidence of candidaemia caused by common species in ICU patients with COVID-19 was higher than patients without COVID-19. Interestingly, the higher incidence was not driven by patient-to-patient *Candida* spp. transmission suggesting that individual risk factors might also contribute to the rising number of *Candida* infections (Machado et al., 2022).

The management of *C. auris*-infected patients is cumbersome since conventional biochemical microbiological techniques are unable to properly identify *C. auris* and isolates are often associated with multi- or pandrug resistance yielding high therapeutic failure rate with all types of antifungal treatments (Chaabane et al., 2019; Fasciana et al., 2020). The relationship between minimal inhibitory concentration (MIC) values and clinical outcomes is still not fully understood, resulting in a lack of established susceptibility breakpoints for *C. auris*. As *C. auris* pose substantial risks for infection control and prevention, the CDC has defined tentative antifungal breakpoints using susceptibility data from hundreds of clinical *C. auris* isolates. Based on tentative breakpoints, echinocandins are recommended as initial treatment for *C. auris* infections and, if no clinical improvement is observed, amphotericin B can be prescribed, or amphotericin B should be added in combination with other antifungal drugs (Table 2; Centers for Disease Control and Prevention, 2020, 2021). Nevertheless, *C. auris* has the capacity to rapidly acquire resistance to antifungals *in vivo*. A report from India showed that multidrug-resistant *C. auris* was responsible for two third of candidaemia cases among COVID-19 patients in New Delhi with a case-fatality rate of 60%. Applying antifungal susceptibility testing, all isolates were resistant to fluconazole (MIC > 32 mg/L) and 30% were non-susceptible to voriconazole (VOR) (MIC > 2 mg/L). Moreover, 40% showed resistance to amphotericin B (MIC > 2 mg/L) and 60% were resistant to 5-flucytosine (MIC > 32 mg/L). Overall, 30% of *C. auris* isolates were multiazole (fluconazole + VOR) resistant, whilst 70% were multidrug resistant (Chowdhary et al., 2020). Another study reported large number of fluconazole resistant clinical isolates (99.8%) in New York. Fifty percent of the isolates were amphotericin B resistant, whereas echinocandin resistance increased from 0 to 4% and pan-resistance increased from 0 to <1% for *C. auris* clinical isolates in a 5-year period (Kilburn et al., 2022). *C. auris* is able to form biofilms, which is also contributed to the increased virulence, antifungal resistance and poor clinical outcomes. Biofilms are structured microbial communities that form on abiotic and biotic surfaces and are embedded in an extracellular matrix influencing drug resistance by hindering drug penetration into dense biofilms. A large number of *C. auris* infections has been connected to the use of health devices that can serve as a source of infection and can spread to other parts of the body (Eyre et al., 2018; Ruiz-Gaitán et al., 2018; Castro et al., 2019; Jabeen et al., 2020). According to these characteristics, *C. auris* is an emerging invasive pathogen in critically ill COVID-19 patients that requires the implementation of strict infectious control measures such as contact precautions, screening, and diligent decolonization of the patients to prevent the potential nosocomial spread.

**TABLE 2:** Recommendations for the management of fungal co-infections in COVID-19 patients.

Fungal infection	First-line treatment	Second-line treatment	Alternative or salvage therapy	References
*Candida auris*	Echinocandins: ANI-loading dose 200 mg, followed by 100 mg/day; or CAS-loading dose 70 mg, followed by 50 mg/day; or MICA-100 mg/day	Liposomal AMB-5 mg/kg/day	Combination regime* against pan-resistant strains (e.g., AMB + flucytosin)	Centers for Disease Control and Prevention, 2021
Aspergillosis	VOR-loading dose 6 mg/kg twice a day, followed by 4 mg/kg twice a day; or ISZ-loading dose 200 mg three times a day for six doses, followed by 200 mg once a day	liposomal AMB-3 mg/kg/day (except for patients with renal insufficiency)	POS or echinocandin + azole (e.g., ANI + VOR)	Koehler et al., 2021
Mucormycosis	surgical debridement of necrotic tissue + liposomal AMB-5 mg/kg/day (in severe cases higher dose, 10 mg/kg/day is recommended)	POS-300 mg twice a day on day 1, followed by 300 mg/day; or ISZ-200 mg on day 1-2, followed by 200 mg/day	POS/ISZ or AMB in combination with POS or ISZ	Cornely et al., 2019; Chao et al., 2022; Hoenigl et al., 2022

ANI, anidulafungin; CAS, caspofungin; MICA, micafungin; AMB, amphotericin B; VOR, voriconazole; ISZ, isavuconazole; POS, posaconazole. *In vitro studies.

## Aspergillosis

*Aspergillus* spp. are ubiquitous, environmental molds, forming spores that enter the body *via* inhalation. *A. fumigatus* is the most common etiological agent worldwide, probably due to the relatively small size of conidia, which allows for its deep penetration into the alveolar space. *Aspergillus* spores that reach the lungs of immunocompetent humans are generally eliminated by cellular components of the innate immune system, such as neutrophils and macrophages (Hohl and Feldmesser, 2007). However, *Aspergillus* spp. can cause a variety of clinical manifestations in immunocompromised individuals. Following inhalation or inoculation with spores that circulate in the environment, infection may develop leading to allergic reactions or to infectious diseases, which may progress from the respiratory system to a disseminated or invasive infection (Latgé and Chamilos, 2019). Invasive pulmonary aspergillosis (IPA) was reported to be common in critically ill patients that cause high morbidity and mortality (Barnes and Marr, 2006). Several factors predispose hospitalized patients to IPA including corticosteroid therapy, antibiotics, and hematologic malignancy. Respiratory viral infections such as influenza have also been associated with IPA (Schauwvlieghe et al., 2018). Due to immunopathological similarities between severe influenza and severe acute respiratory syndrome coronavirus 2 (SARS-CoV-2) pneumonia (e.g., cytokine storm syndrome, epithelial damage within the airways, lymphopenia) critically ill patients are at risk of secondary infections with *Aspergillus* spp. (Khorramdelazad et al., 2021). The diagnosis of COVID-19-associated pulmonary aspergillosis (CAPA) is difficult as COVID-19 patients in ICUs generally have less-specific radiological signs of infection in the presence of acute respiratory distress syndrome (Koehler et al., 2019; Bartoletti et al., 2021). The recently proposed consensus criteria for definition of CAPA facilitate the uniform CAPA classification across medical practices enabling more accurate estimation of aspergillosis cases in COVID-19 patients. Diagnosis of CAPA relies especially on direct microscopic indication of fungal characteristics that are specific of *Aspergillus* spp., culture-based methods and indirect fungal biomarkers (galactomannan and 1,3-β-D-glucan). In accordance with sample validity and diagnostic evidence, proven, probable and possible CAPA categories have been established. Although upper respiratory samples often cannot distinguish between *Aspergillus* colonization and invasive disease, and serum galactomannan as well as serum β-D-glucan exhibit suboptimal sensitivity and specificity, the detection of galactomannan in lower respiratory samples (i.e., bronchoalveolar lavage) is highly suggestive for CAPA (Koehler et al., 2021). Even though the consensus regarded bronchoscopy with bronchoalveolar lavage as an efficient technique in the detection of CAPA, bronchoscopy is not routinely performed in many institutions to reduce COVID-19 transmission risk related to aerosolisation generated during this sampling method (Tio et al., 2021). Therefore, the majority of reported cases were classified as probable or possible CAPA as it has been commonly difficult to prove the correlation between IPA and COVID-19 (Lamoth et al., 2021). A systematic review of autopsy series with histopathological investigations of COVID-19 decedents have also demonstrated the problems with CAPA diagnosis since post-mortem incidence of CAPA was somewhat lower than expected based on clinical findings (Kula et al., 2021).

There are additional risk factors typically connected with the management of severe COVID-19 patients. Prospective and retrospective cohort studies with COVID-19 patients admitted to ICUs showed association between the use of high-dose corticosteroid administration and *Aspergillus* co-infection (Bartoletti et al., 2021; Dellière et al., 2021). Moreover, anti-interleukin-6 (IL-6) receptor treatment, such as tocilizumab therapy that is widely used to treat COVID-19, seems to potentially confer higher risk for developing CAPA as the significantly elevated level of IL-6 in severe COVID-19 patients has also been found as a contributing factor in protection against *Aspergillus* (Table 1; Guaraldi et al., 2020; Bartoletti et al., 2021; Feys et al., 2021). Assessing the worldwide burden of CAPA is complicated due to differences in diagnostic criteria and the wide range of respiratory specimen types obtained for mycological diagnostics. Additionally, observational studies have been performed mainly in European countries and only few data from other continents are available (Feys et al., 2021). Based on analysis of clinical data from several countries, regional variability in CAPA incidence have been observed ranging from 0.54 to 42.1% considering overall CAPA incidence (Feys et al., 2021; Takazono et al., 2021; Jiang et al., 2022). Lahmer et al. (2021) investigated 32 patients with severe COVID-19 associated pneumonia and reported a high incidence of CAPA among COVID-19 patient (11/32; 34%) (Lahmer et al., 2021). Similar incidence rate (8/19; 42.1%) was observed by Jiang et al.; however, both studies were conducted with limited number of COVID-19 patients (Jiang et al., 2022). Despite the differences in reported incidences of CAPA, COVID-19 increased the risk of developing an IPA and CAPA was significantly associated with higher mortality rate (up to 50%) underscoring the importance of global awareness and early diagnosis (Ezeokoli et al., 2021; Lahmer et al., 2021; Meijer et al., 2021; Jiang et al., 2022).

The first-line antifungal agents recommended by the European Confederation of Medical Mycology (ECMM) and International Society for Human and Animal Mycology (ISHAM) for CAPA are VOR or isavuconazole (Koehler et al., 2021). However, several adverse effects related to VOR treatment are known (e.g., liver abnormalities, gastrointestinal disturbance). VOR also has multiple drug-drug interactions including remdesivir, which is generally used in COVID-19 treatment, as both drugs are metabolized by cytochrome P450 enzyme CYP3A4 (McCreary and Pogue, 2020). Plasma concentration monitoring is required due to the unpredictable metabolism of VOR as both subtherapeutic and toxic levels have been detected in critically ill patients. Its narrow therapeutic window and toxicity along with its interactions with other drugs yielded its limited use in ICU patients (Cadena et al., 2021; Tio et al., 2021). Compared to VOR, isavuconazole has better pharmacokinetic profile and is less toxic, but also serves as a substrate for CYP3A4 reducing its efficacy. Liposomal amphotericin B is the alternative option except for patients with COVID-19 related renal insufficiency. Alternative second-line agents are posaconazole or echinocandins. Echinocandins should be used for salvage therapy or in combination with other drugs (Table 2; Koehler et al., 2021).

Another growing concern about the management of CAPA is the case reports of triazole-resistant *A. fumigatus* infections in COVID-19 patients. Triazole-resistance of *Aspergillus* species often varies substantially among geographic regions (from less than 1% in France to an estimated ∼11% prevalence in the Netherlands) worsening the prognosis of patients with invasive aspergillosis (Alanio et al., 2011; Lestrade et al., 2020). Isolates can acquire azole resistance either following prolonged azole treatment of patients in clinical settings by single point mutations in the lanosterol 14-α-demethylase gene (*cyp51A*), encoding a key protein in the ergosterol biosynthesis pathway or by tandem repeat integrations of different sizes in the *cyp51A* promoter inducing point mutations in the gene (TR34/L98H, TR46/Y121F/T289A, and TR53) due to the selective pressure caused by the extended use of demethylation inhibitors in agriculture (Arastehfar et al., 2020b; Garcia-Rubio et al., 2017). Considering CAPA, five cases of triazole-resistant *A. fumigatus* have been reported so far and four out of five isolates had TR34/L98H mutation in *cyp51A* gene, which is associated with acquired environmental resistance, commonly resulting in pan-azole resistance (Borman et al., 2020; Ghelfenstein-Ferreira et al., 2021; Meijer et al., 2020, 2021; Mohamed et al., 2021). This environmentally acquired resistance in *cyp51A* gene is in line with clinical data where most of the patients with azole-resistant infections had no history of azole prophylaxis or treatment (Meis et al., 2016; Ghelfenstein-Ferreira et al., 2021). The occurrence of triazole and multi-triazole resistance underscores the importance of early diagnosis and the urgent need for antifungal drug susceptibility testing of *Aspergillus* isolates on a routine basis using a rapid and simple phenotypic method and/or by detection of *cyp51A* gene associated triazole resistance mutations directly on respiratory samples.

## Mucormycosis

Mucormycosis is an angioinvasive fungal infection caused by filamentous fungi of the order of *Mucorales*. It is the third most common fungal infection after aspergillosis and candidiasis accounting for 9% of all invasive mycosis in immunocompromised patients (Pasternak and Olszanecki, 2021). The disease is characterized by rapidly progressive nature and high mortality rate (reaching 40-80%) even despite adequate treatment. The most frequently identified pathogens responsible for mucormycosis are *Rhizopus* spp., *Mucor* spp., *Lichteimia* spp., *Rhizomucor* spp., *Cunninghamella* spp., *Apophysomyces* spp., and *Saksenaea* spp. (Cornely et al., 2019). The incidence of mucormycosis in recent years and during COVID-19 pandemic has increased gradually, especially in India (Hussain et al., 2021). During the pre-pandemic era, the prevalence of mucormycosis varied from 0.005 to 1.7 per million population worldwide, whereas in India it was 80 times more common with approximately 0.14 cases per 1,000 individuals (Skiada et al., 2020). India have seen an enormous increase in COVID-19-associated mucormycosis (CAM) cases in the second wave of COVID-19 that was declared as an outbreak in May 2021 leading to a collapse of the healthcare system in the middle of the pandemic (Hussain et al., 2021). Nevertheless, the incidence of mucormycosis in patients having or recovering from COVID-19 has been escalating throughout the world. Multiple CAM case reports from United States, Pakistan, Iran, Mexico and single case reports from Brazil and Chile have been published (Khan et al., 2020; Monte Junior et al., 2020; Rabagliati et al., 2021; Shakir et al., 2021; Tabarsi et al., 2021; Guzmán-Castro et al., 2022). In Europe, CAM cases have been reported from Austria, Germany, Spain, Czech Republic, France, Italy, the Netherlands and the United Kingdom (Hanley et al., 2020; Arana et al., 2021; Bellanger et al., 2021; Buil et al., 2021; Pasero et al., 2021; Zurl et al., 2021; Danion et al., 2022; Hoenigl et al., 2022; Seidel et al., 2022).

*Mucorales* are abundant in nature and can be found on decaying organic matter and in the soil. Infection may develop by inhaled fungal spores that are ubiquitously present in the air or by inoculation of the spores into disrupted mucosa or wounds. The hyphal growth cause invasion of blood vessels, resulting in thrombosis and progressive necrosis that leads to soft tissue and bone destruction irrespective of the route of invasion (Pasternak and Olszanecki, 2021; Kamath et al., 2022). In susceptible hosts, six distinctive clinical manifestation could be observed including rhino-orbital-cerebral, pulmonary, gastrointestinal, cutaneous, disseminated, and uncommon infection. Rhino-orbital-cerebral is the most common form of mucormycosis with *Rhizopus* spp. being the most prevalent causative pathogen (Pasternak and Olszanecki, 2021; Hoenigl et al., 2022). The major predisposing factors for the development of mucormycosis are uncontrolled diabetes mellitus with ketoacidosis, neutropenia, hematological malignancy, stem cell and solid organ transplantations, iron chelation therapy with deferoxamine, and corticosteroid usage (Hussain et al., 2021; Hoenigl et al., 2022; Kamath et al., 2022). Hyperglycemic state, low oxygen level, high iron levels, an acidic medium, and a decreased phagocytic activity occurred in most COVID-19 patients favor the growth of fungi, particularly *Mucorales* that use free iron levels in the serum for their pathogenesis (Morales-Franco et al., 2021; Darwish et al., 2022).

The clinical and radiological features of pulmonary and disseminated mucormycosis are non-specific and could overlap with signs associated with COVID-19 making the diagnosis problematic. Additionally, the result of imaging techniques or serological tests from sputum and BAL samples are inconclusive, hence CAM can also be misdiagnosed as CAPA, which is the predominant mould infection in patients with COVID-19-associated acute respiratory distress syndrome (Fathima et al., 2021; Pasternak and Olszanecki, 2021). It is considered that histopathological examination of paraffin-embedded tissue samples is a gold standard for the diagnosis of disease. Species belong to *Mucorales* have broad, ribbon-like, aseptate hyphae branching at right angles. New molecular diagnostic techniques such as polymerase chain reaction (PCR) may offer an alternative approach allowing fast diagnosis which can lead to an early initiation of therapy (Hammond et al., 2011; Darwish et al., 2022). The risk of all-cause mortality of mucormycosis is high (54%) depending on body site infected, fungus type, and the patient’s overall condition. The lack of routinely available fungal biomarker for mucormycosis results in delayed diagnosis; thus, CAM in patients with known co-morbidities (diabetes, transplantation, malignancies) and medications (steroids) has often fatal outcome (Table 1; Monte Junior et al., 2020; Kanwar et al., 2021; Waizel-Haiat et al., 2021). Co-infections with multiple fungal species also worsen the survival rate of COVID-19 patients that was shown in a case report of combined aspergillosis and mucormycosis (Benhadid-Brahmi et al., 2022).

Essential treatment principles of mucormycosis are control of the underlying disease or risk factor, surgical debridement of necrotic infected tissue, and the implementation of adequate antifungal pharmacotherapy. Surgical intervention prior to the spread of infection to different organs and tissues is crucial in the successful management of mucormycosis as it is associated with significantly better clinical outcomes when combined with early, adequate systemic antifungal therapy (Brunet and Rammaert, 2020). In general, patients will be treated empirically for mucormycosis due to the difficulty of correct diagnosis. However, effective treatment options are limited because *Mucorales* are naturally resistant to the majority of antifungal agents causing therapeutic failure (Drogari-Apiranthitou et al., 2012; Darwish et al., 2022). First-line treatment with high-dose liposomal amphotericin B is strongly recommended for serious life-threatening mucormycosis since lipid formulations of amphotericin B have less nephrotoxicity than other formulations that particularly advantageous when given in high daily doses in case of orbital-cerebral involvement. Posaconazole and isavuconazole have emerged as second-line or salvage therapy for individuals with impaired renal function. VOR and echinocandins are ineffective. Other alternative strategies against mucormycosis (e.g., combination therapy) should be considered in severe cases (Table 2; Chao et al., 2022; Hoenigl et al., 2022).

## Peptides With Antifungal Activity as Potential Therapeutic Agents

**FIGURE 1:**
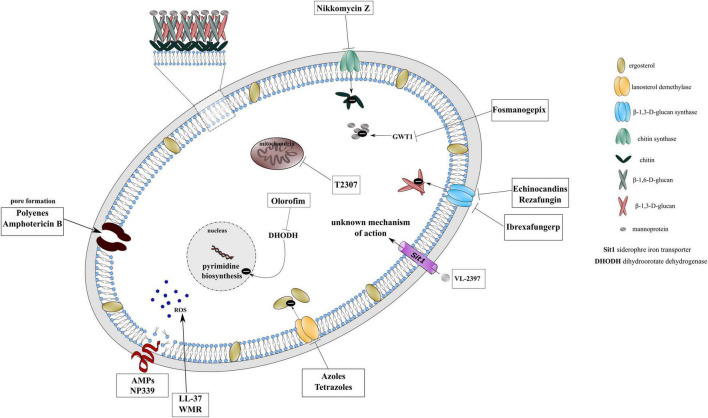
Mechanism of action of conventional antifungal drugs, novel agents under development and potential antimicrobial candidates on cellular targets.

The narrow spectrum of activity, adverse effects, drug-drug interactions of currently used antifungals and the high emergence of resistance has triggered the search for new agents with improved safety profile and broad-spectrum antimicrobial activity. Novel antifungals are being currently developed to overcome these limitations especially reducing their toxicity, optimizing pharmacodynamics and pharmacokinetics, improving their formulations, and increasing specificity. Some developments have been focused on agents with new structure for a known target (rezafungin, tetrazoles, ibrexafungerp) or establish entirely novel targets (fosmanogepix, olorofim) (Figure 1; Gintjee et al., 2020; Bouz and Doležal, 2021). In spite of current efforts, antifungal drug resistance remained a major concern thus introducing alternative therapeutic approaches are irrefutable. One of the alternative treatment strategies might be the use of quorum-sensing molecules (e.g., farnesol, tyrosol) or non-antifungal agents in combination with traditional antifungals (Kovács and Majoros, 2020; Rossato et al., 2021). Other potential antimicrobial candidates attracting the attention of researchers are antimicrobial peptides (AMPs). AMPs are host defense peptides playing essential role in innate immune response. AMPs are composed of 15–50 amino acids that are mainly cationic at physiological pH and their amphipathic conformation facilitates the interaction with the negatively charged membrane of microorganisms leading to membrane insertion, destabilization and disruption of the cell (Figure 1). Resistance to AMPs is less likely to emerge because AMPs demonstrate rapid and drastic effect on fungal cell membrane that otherwise evolves slowly (Fernández de Ullivarri et al., 2020; Mookherjee et al., 2020).

The knowledge of antifungal effect of modified AMPs falls away that for the antimicrobial activity of AMPs against pathogenic bacteria, however, some new antifungal peptides offer possible therapeutic alternatives. Duncan et al. evaluated the antifungal activity of a 2-kDa polyarginine peptide (NP339) obtained by a solid-phase synthesis, which was inspired by host defense peptides (Duncan et al., 2021). NP339 showed pronounced antifungal activity against *C. albicans* as rapid cell destruction was seen after 4 mg/L NP339 (1xMIC) exposure with electron microscopy analysis within 30 min. In contrast, caspofungin and human β-defensin 2 did not demonstrate similar effect after exposure of 1xMIC concentration. NP339 was also active against *Aspergillus* species including *A. fumigatus, A. flavus*, and *A. niger* by lysing biofilm forming cells. Time-kill experiments with *C. auris* revealed same effectiveness of NP339 than amphotericin B and it was more effective than fluconazole and caspofungin. Further advantage of this molecule is that no cytotoxic effect was observed on human peripheral blood mononuclear cells and the A549 epithelial lung cell line even at the concentration of hundreds of folds higher than the proposed therapeutic dose. Additionally, exogenous peptides are not metabolized in the liver reducing the risk of drug-drug interactions. Although authors did not notice significant effect of NP339 administration in murine model of disseminated candidiasis, nebulized NP339 reduced the fungal burden of the lungs in rodents with invasive pulmonary aspergillosis. Of note, the method of kidney fungal burden evaluation is not optimized for membrane-acting agents since during tissue homogenization the peptide-fungal cell interaction will be disbanded consequently yielding underestimation of peptide activity.

LL-37 is a human cathelicidin-related peptide produced by macrophages, neutrophils, various epithelial cells and natural killer cells. It interacts with cell wall carbohydrates and destabilizes membrane permeability with elevating reactive oxygen species (ROS) causing oxidative stress within the cell. LL-37 proved to be fungicidal against *C. auris* including resistant or multidrug resistant strains and found synergy when combined with fluconazole in most of the *C. auris* strains (80%), whereas, synergism was also observed in the presence of LL-37 with amphotericin B and caspofungin in all *C. auris* strains. Cell count and viability assay was performed as well in order to confirm the fungicidal potential of LL-37. Based on results, LL-37 inhibited the growth and survival of *C. auris* cells. Nevertheless, further *in vivo* investigations are needed regarding LL-37 cytotoxicity on mammalian cells to be a potential drug candidate for combating *C. auris* infections (Rather et al., 2022). Unfortunately, controversial results have been reported for LL-37 activity in case of *Aspergillus* species (Luo et al., 2019; Ballard et al., 2020), however, cathelicin-based synthesized peptides seem to be promising antifungals against *A. fumigatus* (van Eijk et al., 2020).

Myxidin, originated from the epidermal mucus of hagfish (*Myxine glutinosa* L.), was modified (WMR) by Maione et al. (2022) to increase the number of positively charged amino acids in the original sequence. The peptide previously represented antimicrobial activity against Gram-positive and Gram-negative bacteria. According to this activity, *in vitro* and *in vivo* studies were carried out to determine the antifungal effect of WMR. Studies tended to investigate WMR mechanism of action revealed that it induces ROS generation resulting cell death *via* oxidative damage. Although WMR exhibited low antifungal activity for *C. albicans, C. auris*, and *C. glabrata* planktonic cells, it had significant anti-biofilm effect by eradication of mature biofilms formed by *C. tropicalis, C. parapsilosis*, and *C. auris*. The synergism in combination with fluconazole also promotes the consideration of possible therapeutic application of WMR. Bugli et al. designed lipopeptides starting from the sequence of the amphipathic α-helix of chionodracine by fatty acid acylation with myristic acid (Olivieri et al., 2018; Bugli et al., 2022). Myristoylated peptides showed lower MIC_90_ values against *C. albicans, C. glabrata, C. parapsilosis, C. tropicalis*, and *C. auris* than the native peptides with outstanding antifungal activity of Myr-B against *C. auris*. Myr-B seemed to be effective 24 h post-infection against multidrug-resistant *C. auris* in *Galleria mellonella* model as well at a concentration of 640 μg/mL and was not toxic when testing on primary human fibroblast cell line (FB789).

## Future Perspective

Secondary fungal infections associated with COVID-19 are an emerging major concern as delayed diagnosis due to similar symptoms of infections and difficulty of identification methods and treatment increase the mortality rate in ICUs especially in patients with underlying diseases. Besides candidiasis, aspergillosis and mucormycosis; fungaemia caused by *Cryptococcus* and *Trichosporon* species are also increasingly reported in COVID-19 patients (Ali et al., 2021; Gil et al., 2021). Rapid and reliable identification of fungal pathogens and implementation of strict infection control strategies challenges the medical practices resulting underestimation of fungal co-infection in COVID-19 patients. The dosage and duration of treatment with corticosteroids, immunosuppressants and broad-spectrum antibiotics should be considered carefully as well. Prompt diagnosis along with adequate antifungal management may improve the survival rate of hospitalized COVID-19 patients with fungal co-infections, however, the available therapeutic options are limited and the emergence of resistant or MDR fungal species impels us to search for new alternatives to overcome nosocomial drug-resistant infections in hospital settings. The diversity and broad-spectrum activity of AMPs qualify them as promising candidates for the development of novel antimycotics. The poor pharmacological properties, toxicity and high cost in large-scale production hampers the commercial applications of AMPs. In spite of these disadvantages, there are some promising AMPs currently in pre-clinical or clinical trials including NP339 (Novamycin) and LL-37. Of note, LL-37 involved in the treatment of leg ulcers and it has been not tested against fungal infections (Koo and Seo, 2019). Although further investigations on recently published AMPs are required regarding their distinct mechanism of action and toxicity, modified natural AMPs will be involved in clinical setting in the future facilitated by the rapid development of *in silico* analyses that help the optimization of peptides.

## Author Contributions

MD: conceptualization, data collection, and writing the draft manuscript. KB: data collection and revising and editing the manuscript. Both authors have agreed to the published version of the manuscript.

## Conflict of Interest

The authors declare that the research was conducted in the absence of any commercial or financial relationships that could be construed as a potential conflict of interest.

## Publisher’s Note

All claims expressed in this article are solely those of the authors and do not necessarily represent those of their affiliated organizations, or those of the publisher, the editors and the reviewers. Any product that may be evaluated in this article, or claim that may be made by its manufacturer, is not guaranteed or endorsed by the publisher.
